# Acute kidney injury biomarkers: renal angina and the need for a renal troponin I

**DOI:** 10.1186/1741-7015-9-135

**Published:** 2011-12-21

**Authors:** Stuart L Goldstein

**Affiliations:** 1 University of Cincinnati College of Medicine, Center for Acute Care Nephrology, Division of Nephrology and Hypertension, The Heart Institute, Cincinnati Children's Hospital Medical Center, 3333 Burnet Avenue, MLC 7022 RILF2, Cincinnati, OH, 45229 USA

## Abstract

Acute kidney injury (AKI) in hospitalized patients is independently associated with increased morbidity and mortality in pediatric and adult populations. Continued reliance on serum creatinine and urine output to diagnose AKI has resulted in our inability to provide successful therapeutic and supportive interventions to prevent and mitigate AKI and its effects. Research efforts over the last decade have focused on the discovery and validation of novel urinary biomarkers to detect AKI prior to a change in kidney function and to aid in the differential diagnosis of AKI. The aim of this article is to review the AKI biomarker literature with a focus on the context in which they should serve to add to the clinical context facing physicians caring for patients with, or at-risk for, AKI. The optimal and appropriate utilization of AKI biomarkers will only be realized by understanding their characteristics and placing reasonable expectations on their performance in the clinical arena.

## Background

Acute kidney injury (AKI) is defined as an abrupt decrease in kidney function, which in its most severe form, acute renal failure, is manifested by changes in blood chemistry and decreased urine output [1]. AKI rates among hospitalized adults and children have been rising over the past two decades. Discharge coding data from a 5% sample of United States Medicare beneficiaries (n = 5.4 million) demonstrated an 11% annual increase in AKI prevalence in hospitalized adults between 1992 and 2001 (the prevalence increased from 14.2 to 34.6 AKI cases per 100 patient discharges) [2]. AKI rates likewise increased 20-fold, from 0.5 to 9.9 cases per 1,000 hospitalized children, between 1982 to 2004 [3].

The AKI landscape has undergone a seismic shift in the last ten years. Prior to 2004, the definition of AKI was not standardized, with more than 30 different definitions used in the published literature [4]. Rigorous assessment of cross-sectional and longitudinal epidemiology of any condition requires a standard definition. The reassessment of AKI epidemiology with a focus on organ crosstalk [5-7], standardization of the AKI definition [4,8,9], collaborative multi-center continuous renal replacement therapy (CRRT) research [10-12], and recognition of chronic kidney disease (CKD) development in AKI survivors [13], all point to a renewed understanding that AKI is far from a benign syndrome. In fact, the realization that patients are dying 'from' and not just 'with' AKI [14], and that pre-renal azotemia may not be a benign disease state [15], have created the impetus to prevent or mitigate the effects of AKI. However, clinicians caring for patients with AKI have been hindered by the reliance on serum creatinine or decreased urine output, both kidney function markers, to make the AKI diagnosis. In the setting of acute tubular necrosis (ATN), these functional changes only manifest after significant kidney damage has taken place. Even more vexing is the fact that similar serum creatinine changes can occur without kidney damage, for example in the context of dehydration, nephrotic syndrome or hepatorenal syndrome. In light of the independent association between AKI and mortality rates of up to 60% in critically ill patients and our current ability to only provide supportive care for patients with AKI, the need for more precise and earlier diagnostic tools is profound.

Optimal therapeutic interventions require expeditious diagnosis for any disease state. The advancements in cardiac and oncological treatments over the past decades have in large part been enabled by the discovery, validation and implementation of new biomarkers of disease; these have included advanced imaging techniques as well as specific markers of cardiac myoblast injury or the genetic subtypes of specific cancers. The myocardial ischemia diagnostic paradigm has moved from electrocardiographic changes, to creatine phoshopkinase (CPK) measurement, to specific CPK subtype enzymatic changes, to the troponins and now to brain natriuretic peptide (BNP). As a result, the sensitivity and specificity to detect earlier myocardial ischemia has progressively increased, directing earlier intervention that has transformed the field and substantially decreased patient mortality [16,17].

Extensive research efforts over this past decade have been directed at the discovery and validation of novel AKI biomarkers to detect injury prior to changes in kidney function and potentially to aid in the differential diagnosis of AKI. The quest for such biomarkers has often been referred to as the 'search for the renal troponin I'. The analogy to troponin I and its acceptance for prompt evaluation and therapeutic intervention in at-risk patients with the clinical presentation of chest pain is an informative and potentially applicable model to the AKI field [18]. Nephrologists and intensivists must define a 'renal angina syndrome' to initiate optimal assessment with AKI biomarkers to realize their full potential to improve patient care and outcomes.

The purpose of this article is to review the relevant AKI biomarker literature in terms of a contextual framework to aid in the clinical diagnosis of AKI prior to changes in kidney function. In addition, the empiric prodrome of 'renal angina' will be discussed to highlight the need to direct AKI biomarker assessment only where it will optimize clinical care by detecting AKI early, refining AKI differential diagnosis and/or providing information regarding AKI severity. Finally, very recent data demonstrate that AKI biomarker 'positive' but serum creatinine 'negative' AKI has an equally poor prognosis with classical functional AKI [19], suggesting that we may be on the verge of a new, more targeted definition of AKI based on novel biomarkers.

### Serum creatinine - the gold standard is a late marker of kidney injury

AKI detection and differential diagnosis has been mired in reliance on the same functional marker, serum creatinine, used to diagnose chronic kidney disease. Unfortunately, serum creatinine concentrations do not change until significant injury and loss of nephron function has occurred. Chertow and colleagues demonstrated that a serum creatinine rise of ≥ 0.3 mg/dl in hospitalized adults was independently associated with a four-fold increased risk of mortality, even when controlling for diabetes, advanced heart failure, ethnicity and age [20]. Multiple studies using the RIFLE (Risk, Injury, Failure, End-Stage Renal Disease) or Acute Kidney Injury Network criteria in more than 550,000 patients demonstrated that a 50% rise in serum creatinine is associated with adult patient mortality [21]. Similar data have been published in children with acute decompensated heart failure, showing that a ≥ 0.3 mg/dl rise in serum creatinine is independently associated with death or the need for mechanical ventricular assistance [22]. Since such small rises in serum creatinine already reflect independent morbidity and mortality risk, the need for earlier true markers of AKI has become critical.

### AKI biomarkers - the road to validation

An ideal AKI biomarker should be accurate, reliable, easy to measure with a standard assay, non-invasive, reproducible and sensitive and specific with defined cutoff values [23]. Urine represents an ideal body fluid for AKI biomarker assessment as it can be obtained non-invasively and repeatedly from a spontaneously voided sample or from an indwelling bladder catheter. The road to AKI biomarker validation spans discovery in pre-clinical studies from bodily fluids, assay development, retrospective study in completed trials and then prospective screening in ongoing trials [23]. These phases must be completed before a biomarker can be used broadly in clinical practice. Four AKI biomarkers, neutrophil gelatinase-associated lipocalin (NGAL), interleukin 18 (IL-18), Kidney Injury Molecule -1 (KIM-1) and Liver Fatty Acid Binding Protein (L-FABP) have been tested to various degrees in ongoing clinical trials [24].

### Putting AKI biomarkers to the test: the paradigm of AKI after cardiopulmonary bypass

Most early AKI biomarker validation studies have occurred in patients after cardiopulmonary bypass [25-27] or renal transplantation [28]. The reasons for selecting these populations are apparent: the timing of kidney injury is known, biomarkers can be assessed repeatedly after the event, and the AKI event rates after these procedures are well documented. In addition, many early trials assessed AKI biomarkers in children, since they do not have many of the co-morbidities (chronic kidney disease, diabetes, chronic inflammatory diseases) that could potentially confound AKI studies. As a result, one could reasonably argue if an AKI biomarker does not demonstrate adequate sensitivity and/or specificity for predicting AKI development, severity or duration in a child after cardiopulmonary bypass (CPB), then it would be unlikely to perform well in the adult population.

The initial prospective human study of urinary NGAL occurred in the pediatric CPB setting [25]. Seventy-one children were enrolled and AKI was defined as a serum creatinine rise of 50% over baseline. Urinary NGAL was detected and rose 100-fold 2 hours after CPB was initiated in the 21 patients who ultimately developed AKI, whereas NGAL did not rise in the urine of patients who did not develop AKI. Most importantly, serum creatinine concentrations did not increase in AKI patients until 36 to 48 hours after the NGAL increase, which provides a heretofore unavailable potential therapeutic window to intervene and prevent or mitigate AKI.

Subsequently, multiple studies have confirmed the predictive ability of NGAL in the pediatric CPB setting [29-31]. In addition to NGAL, IL-18, KIM-1 [32] and L-FABP [27] have been studied in the post-CPB setting. Very recent data from the large US National Institutes of Health (NIH)-funded multi-center Translational Research Investigating Biomarker Endpoints in Acute Kidney Injury (TRIBE-AKI) consortium has also demonstrated both urinary NGAL and IL-18 [26] in children [33] and adults [34] after CPB. In both populations, NGAL and IL-18 demonstrated moderate predictive ability for AKI with a significant improvement above clinical risk factors alone. The TRIBE-AKI experience has set the standard for AKI biomarker assessment and statistical analysis. Additionally, the consortium plans to follow survivors longitudinally to assess for development of chronic kidney disease. Finally, one very recent study has evaluated the complete 'panel' of these four AKI biomarkers in the pediatric CPB setting [35]. In this study, we demonstrated a temporal pattern of biomarker elevation, with NGAL elevated at 2 hours, IL-18 and L-FABP elevated at 6 hours and KIM-1 elevated at 12 hours in patients who developed AKI after CPB initiation. As with the TRIBE-AKI cohort, urinary biomarkers improved the predictive ability for AKI compared with clinical risk factors alone. This discovery of a temporal pattern argues for the combination of biomarkers in a panel to allow for more precise assessment of the time course of AKI, which would theoretically inform clinical trials in terms of the timing of intervention.

### Validation in other populations

Subsequent to the initial promising results observed for novel biomarker prediction of AKI development and severity after CPB, biomarkers (especially NGAL [36]) have been assessed in multiple other clinical AKI settings including contrast induced nephropathy [37,38], hemolytic uremic syndrome (HUS) [39], lupus nephritis [40], and renal [28,41,42] and orthotopic hepatic transplantation [43]. Once again, AKI biomarkers should be validated in these disease states as they contain either a known timing or mechanism of injury (nephrotoxins or surgery), or they represent a primary acute kidney disease (lupus nephritis, HUS, kidney transplantation).

In addition, other non-surgical or non-kidney specific disease states such as acute decompensated heart failure-associated cardiorenal syndrome may provide an excellent arena to study novel AKI biomarkers [17]. The use of biomarkers in other systemic illnesses with unknown timing of insult, such as septic shock or critical illness, represents a major challenge for biomarkers to predict AKI development and/or severity. Our initial work in critically ill children receiving invasive mechanical ventilation and at least one vasoactive medication demonstrated that NGAL [44] and IL-18 [45] could predict ultimate AKI severity by the pRIFLE score and duration of AKI. In addition, NGAL rose two days prior to serum creatinine in all patients who developed AKI, and IL-18 rose 2 days prior to serum creatinine in non-septic patients who developed AKI. Recent biomarker data from adults measured in the ICU demonstrated reasonable performance of NGAL, IL-18 and Cystatin C to predict AKI when stratified by baseline estimated glomerular filtration rate (eGFR) and at different time points within the first two days of ICU stay [46].

### The next phase for AKI biomarkers

All of the AKI biomarker work performed to date has focused on discovery and validation of AKI biomarkers in a post-hoc manner; samples for biomarkers have been obtained and stored for later assessment of their ability to predict AKI development or severity. The next challenge for AKI biomarkers is to test their ability to direct therapeutic intervention or other clinical management. Yet, the heterogeneity of patient populations and varying precision of AKI biomarkers noted above presents a significant risk for inappropriate use of AKI biomarkers to decrease their utility. Recent statistical methods such as the net reclassification index must be used to ensure that biomarker concentration thresholds add predictive value to the clinical model alone in predicting the AKI spectrum. A recent concept of a prodome of 'renal angina' has been proposed to direct biomarker assessment only in patients who fulfill a combination of illness severity/risk and small changes in kidney function (creatinine changes or fluid overload) [18,47]. The renal angina concept is based on the high negative predictive value of the construct; patients who do not fulfill renal angina should not have biomarker assessment as their risk of developing AKI is extremely low. However, renal angina presence only increases AKI development risk and biomarkers should add value to predict the AKI spectrum and help guide management.

## Conclusions

As discussed above, AKI biomarkers have been validated retrospectively in multiple patient populations. The challenge for the future is to use these data to design preventive, interventional and supportive clinical studies to test the value of AKI biomarkers in improving the outcome for patients with, or at-risk for, AKI. Only after AKI biomarkers have been validated prospectively in the appropriate populations will widespread and rational adoption be possible.

## Abbreviations

AKI: acute kidney injury; ATN: acute tubular necrosis; BNP: brain natiuretic peptide; CKD: chronic kidney disease; CPB: cardiopulmonary bypass; CPK: creatine phosphokinase; CRRT: continuous renal replacement therapy; eGFR: estimated glomerular filtration rate; HUS: hemolytic uremic syndrome; IL-18: interleukin-18; KIM-1: kidney injury molecule-1; L-FABP: liver-type fatty acid binding protein; NGAL: neutrophil gelatinase-associated lipocalin; RIFLE: risk; injury, failure, loss, end-stage kidney disease

## Competing interests

Dr. Goldstein has no competing interests to declare with respect to this article.

## Pre-publication history

The pre-publication history for this paper can be accessed here:

http://www.biomedcentral.com/1741-7015/9/135/prepub
